# Epigenomic biomarkers of cardiometabolic disease: How far are we from daily practice?

**DOI:** 10.1186/s12933-024-02497-4

**Published:** 2024-11-07

**Authors:** Ram Abou Zaki, Ronald C. W. Ma, Assam El-Osta

**Affiliations:** 1 Epigenetics in Human Health and Disease Program, Baker Heart and Diabetes Institute, Melbourne, VIC Australia; 2 School of Translational Medicine, Department of Diabetes, Monash University, Melbourne, VIC Australia; 3 Baker Department of Cardiometabolic Health, The University of Melbourne, Melbourne, VIC Australia; 4 Department of Medicine and Therapeutics, The Chinese University of Hong Kong (CUHK), Sha Tin, Hong Kong SAR China; 5 Hong Kong Institute of Diabetes and Obesity, CUHK, Sha Tin, Hong Kong SAR China; 6 Li Ka Shing Institute of Health Sciences, CUHK, Sha Tin, Hong Kong SAR China; 7 School of Biomedical Sciences, CUHK, Sha Tin, Hong Kong SAR China; 8 Faculty of Health, Department of Technology, Biomedical Laboratory Science, University College Copenhagen, Copenhagen, Denmark

## Abstract

Determining whether someone has cardiometabolic disease (CMD), especially in the early stages, can be complicated. Risk stratification ordinarily depends on an extended process relying on medical history that typically considers blood pressure, cholesterol, smoking and diabetes status. Physicians have long relied on these key patient characteristics to assess CMD risk. However, these widely used clinical assessments are often identified later in life and by definition, in those individuals with progressed disease. This is partly because the onset of CMD naturally occurs in adulthood, however, the underlying processes can occur much earlier in life, even in the absence of obvious symptoms. For one thing, the pathways towards pathology may exist for years before symptom onset. Thus, among other things, there are opportunities to provide doctors with better insights into future disease prediction especially in younger adults with diabetes. The rapid rise in CMD together with the increased rates of obesity and diabetes in this population only emphasises the importance of predictive molecular biomarkers. One notable aspect is that traditional risk scores, such as those based on cholesterol measurements, are frequently found to be within normal ranges in younger populations. At the same time, given the significant overlap in risk factors for cardiovascular disease (CVD) and diabetes, the unmet clinical need is for early biomarkers of CMD that may help improve risk assessment in younger adults. This editorial highlights advances in the use of polygenic risk scores and emerging utility of genetic biomarkers to define intermediate CMD phenotypes discussing new classification criteria involving DNA methylation of genes to improve risk assessment. CMD is the number one cause of mortality and accounts for 31% of all global deaths. CMD is also multifactorial, comprising cardiovascular disease (CVD) and diabetes that have significant overlap in risk factors and disease biology. Diabetes is arguably the strongest risk factor for CVD development. Accounting for almost 90% of diabetes cases worldwide, type 2 diabetes (T2D) affects about 527 million people. The global economic burden is estimated at 1.3 trillion USD annually and is close to 1.8% of global GDP [1]. Despite the progress in preventive and therapeutic measures of CVD, the increasing CMD rates only underscore the important need of molecular biomarkers for early detection [2]. Determining whether someone has CMD usually involves an extended diagnostic process that has become essential for risk stratification and disease prevention [3]. While the onset of CMD typically occurs in adulthood, disease development commences much earlier, and this has scientists questioning whether molecular biomarkers could improve current prognostic risk scores. Predicting which people with T2D are most likely to develop CVD remains a significant challenge despite the recent advances in genetic mapping.

**Graphical abstract:**

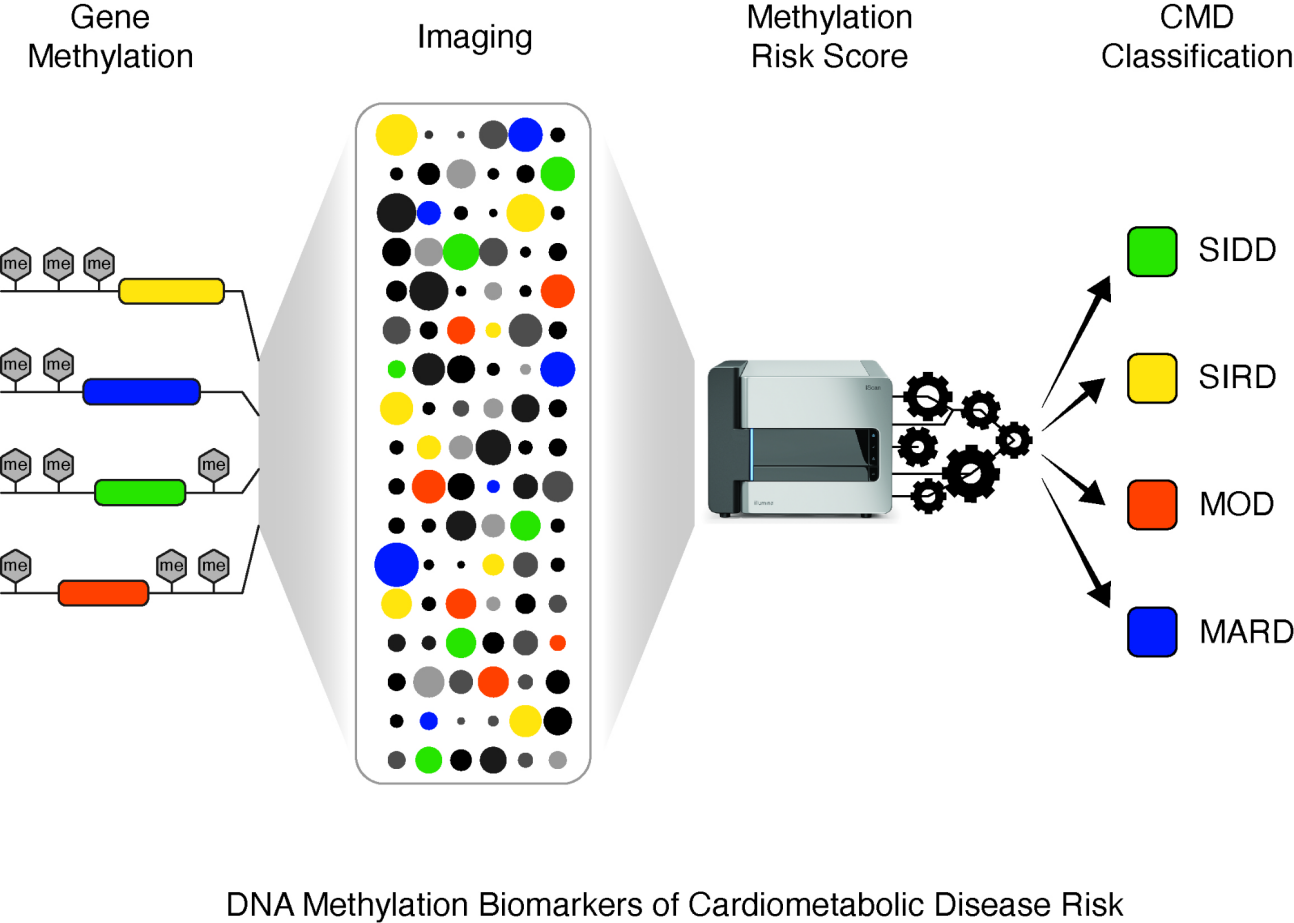

## Epigenetic reclassification of diabetes for precision medicine

Recognising that most genetic biomarkers have a modest predictive value has clinicians reconsidering the widely used CMD risk index [4]. This is an acknowledgement that, unlike age, smoking, low density lipoprotein (LDL) or body mass index (BMI), genetic predisposition in and of itself is not an explicit risk factor of CMD, that is - unless we reassess genetics differently. Recent research has focused on the heterogeneity of diabetes, leading to the identification of distinct subgroups [5]. These subgroups have been identified using unsupervised clustering analysis of clinical factors such as age, BMI, HbA1c at diagnosis, and measures of insulin resistance and beta-cell function [6]. Additionally, prospective follow-up studies have examined the varying risks of complications associated with diabetes, including cardiovascular disease (CVD), chronic kidney disease (CKD), and retinopathy. A biomarker study investigated the links between DNA methylation, or methylation risk scores (MRS) and the four subgroups of type 2 diabetes, and whether DNA methylation can be used to identify those people with T2D at most risk of developing complications [7]. A rigorous examination of differential methylation as an early determinant of disease prompted the team to delineate specific patterns of differential gene methylation that could predict future complications associated with diabetes. It’s that overhaul of T2D groups and redesign in estimating predictive MRS which makes this novel. For the first time, the reclassification also takes kidney function and retinopathy into account by calculating MRS when predicting risk. This article focuses on this epigenetic advancement.

## DNA methylation biomarkers of CMD risk

T2D groups were defined as (i) severe insulin-deficient diabetes (SIDD), (ii) severe insulin-resistant diabetes (SIRD), (iii) mild obesity-related diabetes (MOD), and (iv) mild age-related diabetes (MARD). This reassignment reflects the underlying pathogenesis [7]. For example, people with SIDD generally have higher HbA1c levels because of insulin deficiency, whereas people with SIRD were associated with high HOMA2-IR (homeostatic model assessment of insulin resistance) including high HOMA2-B (beta-cell function) calculated from fasting insulin and glucose levels measurements. The third subgroup MOD was defined by a high BMI at young age, whereas the fourth group, MARD, was correlated with older age. This reclassification improves performance by integrating biological information from common clinical indices such as HbA1c, Age, BMI and HOMA. From their analysis, the investigative team identified DNA biomarkers that were assigned to methylation risk scores (MRS) for the T2D subgroups. MRSs not only distinguish T2D subgroups but could also predict CVD risk. Indeed, this reclassification showed MRS persisted even after adjusting for clinical variables except for SIDD. Genes assigned by MRS were functionally associated with insulin secretion, beta-cell function, obesity, and aging. For example, genes that were differentially methylated and associated with SIDD included *AATK*, *CPLX1*, *CTDSPL*, *GRK5*, *LMNB2*, *RREB1*, *SMARCA4*, *SOD3*, *SYT2*, and *TXNIP* [7]. The expression of these genes are known to influence pathways involved in the secretion of insulin and regulate beta-cell function. Among genes involved with SIRD-MRS, the expression of *RAB27B* and *RBL2* are known to be involved in the regulation of glucose uptake in peripheral organs, along with insulin secretion [8]. Under this framework, it is to be expected that genes associated with obesity—such as *ELOVL2, PDGFC, SCN9A, SLC6A4,* and *TFEB*—were found to be linked to the MOD-MRS.For example, *SCN9A* is known to control satiety and hunger centres in the brain, while other genes regulate fatty acid metabolism emphasising their close correlation with body weight and BMI. This reclassification also goes on to observe differential methylation of genes associated with the aging process such as *CRMP1* and *RNF170* also converge with MARD-MRS. Nevertheless, projects of this nature enhance the classification of CVD in T2D. (Table 1).

**Table 1:** T2D subgroups classification along with the associated clinical phenotypes. Genes were identified using the MRS and include general function.

T2D subgroup	Phenotype	Genes by MRS	Gene Associations
**SIDD**	**High HbA1c**	***AATK***,*** CPLX1***,*** GRK5***,*** LMNB2***,*** RREB1***,*** SOD3***,*** SMARCA4***,*** SYT2***,*** TXNIP***	**beta-cell secretion**
**SIRD**	**HOMA**	***RAB27B***,*** RBL2***	
**MOD**	**High BMI**,** early age of onset**	***ELOVL2***,*** PDGFC***,*** SCN9A***,*** SLC6A4***,*** TFEB***	**Obesity**
**MARD**	**Older age**	***CRMP1***,*** RNF170***	**Aging**

Over a mean follow-up of 4.5 years, higher SIDD-MRS (hazard ratio [HR] 0.72, *P* = 0.032) and MOD-MRS (HR 0.65, *P* = 0.001) were associated with a lower risk of developing CVD. In contrast, a higher SIRD-MRS (HR 1.47, *P* = 0.002) and MARD-MRS (HR 1.41, *P* = 0.007) were linked with elevated risk of future CVD. The researchers found that people with higher SIRD-MRS and MARD-MRS were more likely to develop CVD complications when compared to those people with higher SIDD-MRS and MOD-MRS. The findings of the study also underscore the need to consider obesity in these models, as it’s a known risk factor of metabolic syndrome and vascular complications. In addition to the predictive value of MRS, a correlation was also observed with the development chronic kidney disease. People with higher SIRD- or MARD-MRS were at increased risk for chronic kidney disease (CKD). In contrast, those people with higher MOD-MRS had a decreased risk of CKD. Importantly, these results remained significant after adjusting for blood cell composition but did not reach an AUC above 0.75, suggesting further opportunities to improve sensitivity.

**Fig. 1:**
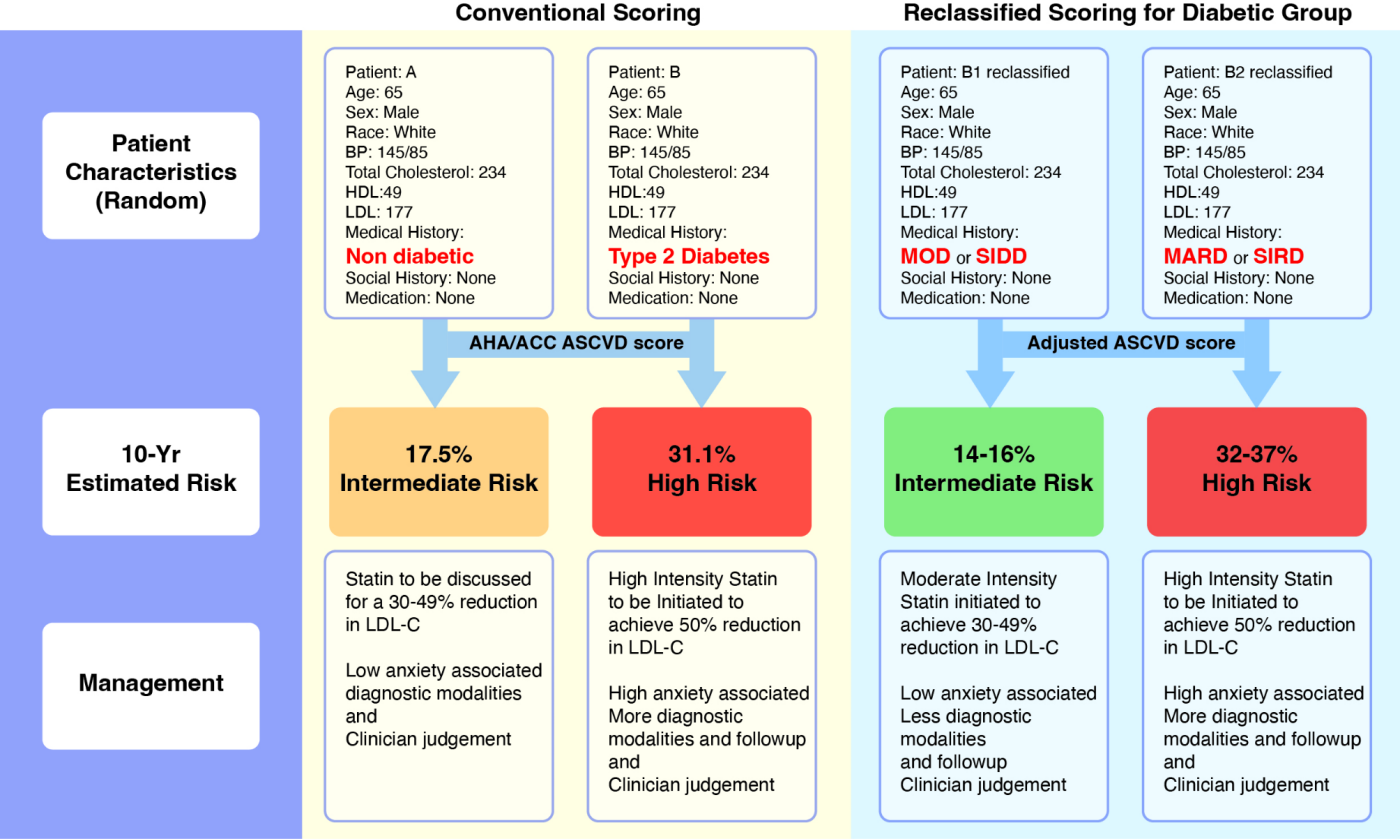
Conventional scoring system and a hypothetical reclassified scoring system based on T2D subgroups: severe insulin-deficient diabetes (SIDD), severe insulin-resistant diabetes (SIRD), mild obesity-related diabetes (MOD), and mild age-related diabetes (MARD). Risk of cardiovascular disease is calculated using ASCVD 10-year risk estimator for the conventional scoring. For the suggested adjusted scoring system, individuals are reclassified depending on the diabetic subgrouping rather than accounting for T2D as one entity. Furthermore, accounting for T2D subgrouping changes the risk of CVD and impose changes on the patient therapeutic plan and disease burden on nations. Considering the newly discovered subgroups will refine the approach and outcomes of patients living with T2D and CVD.

Clinical approaches in managing cardiovascular diseases rely on conventional score calculation which doubles the risk of CVD in people with T2D. Well-established scoring systems include the American Atherosclerotic Cardiovascular Disease (ACVSD) and Systematic Coronary Risk Evaluation 2 (SCORE2) charts endorsed by the American College of Cardiology/American Heart Association (AHA/ACC) and the European Society of Cardiology (ESC) [9, 10]. We hypothesise that including MRS into the equation improves CVD prediction using the reclassified T2D subgroups. While SIRD MARD raise the risk of CVD in line with conventional knowledge, MOD and SIDD emphasize decreased risks when compared to traditional scoring methods outlined by the AHA/ACC and ESC guidelines (Fig. 1).

This revision in CVD scoring shows high confidence of the MRS over conventional markers such as HOMA and BMI. Specifically, MRS for SIRD and MOD exhibited higher area under the curve (AUC) values (0.644 and 0.651, respectively) when compared to HOMA-IR (0.490) and BMI (0.587) respectively. Although there might be considerable criticism regarding the recorded AUC values, we believe that this issue is, in fact, temporary. Deep sequencing technologies have now overcome the constraints of the BeadChip array. This raises its real function, using MRS to predict disease years before symptom onset. Drawing closer to improving advice and treatment to those people with T2D, clinicians recognize that CVD is multifactorial and polygenic.

## MRS converge seemingly dissimilar genes and connect common pathways

The high burden of CMD is simply not genetics, whilst genetic risk scores can differentiate risk of Chronic heart disease (CHD), the static nature of risk prediction using genetic risk scores have their limitations. Genetic variability alone cannot account for why some individuals with type 2 diabetes (T2D) experience a disproportionate burden of complications, while others remain free of complications. Differential methylation suggests one of two important considerations. Firstly, the reclassification is neatly tied in with CMD polygenicity, and methylation closely agrees with genes involved in insulin signalling, obesity and aging. Secondly, the investigators’ reassignment of clinical groups relies on DNA methylation as a stable yet dynamic and potentially reversible indices. Implicit in all this is the relevance of gene and environment interactions in CMD. We consider the MRS have the potential to identify those at risk of CHD that can be automated for high throughput processing of methylation measurement with conservative estimates costing $US30-50 per sample [11].

To conclude, predictive biomarkers in cardiovascular disease were long thought of as a too far-out prospect to solve, they are now resolutely closer to defining gene methylation risk scores that form a picture of a clearly complicated puzzle. These methylation studies support a reclassification of T2D aimed at prevention strategies of subgroups and more targeted care for those living with diabetes. Because DNA methylation distinguish between T2D subgroups and associated with future risk of CVD and CKD, the development of these biomarkers is likely to improve patient treatment and prevent the development of diabetic complications.

## Data Availability

No datasets were generated or analysed during the current study.
